# Frequency and Risk Factors for Secondary Malignancies in Patients with Mycosis Fungoides

**DOI:** 10.4274/tjh.2017.0234

**Published:** 2017-12-01

**Authors:** Fatma Pelin Cengiz, Nazan Emiroğlu, Nahide Onsun

**Affiliations:** 1 Bezmialem Vakıf University Faculty of Medicine, Department of Dermatovenereology, İstanbul, Turkey

**Keywords:** T-cell neoplasms, Non-Hodgkin lymphoma, Oncogenes, T-cell mediated immunity

## To The Editor,

Mycosis fungoides (MF), the most common form of cutaneous T-cell lymphoma (CTCL), has an incidence of 6.4 per million people [1]. Patients with CTCL have an increased risk of the development of secondary malignancies, particularly lymphomas [2 ,3]. We conducted a 20-year population-based cohort study to assess the risk factors of secondary cancers in MF patients from our center.

From 1998 to 2015, a total of 143 cases of CTCL were documented in our database. In this same time period, 13 cases (9.1%) of secondary malignancy excluding non-melanoma skin cancer were diagnosed at least 3 months following the diagnosis of CTCL (Table 1). MF patients were grouped by their tumor stage from I to IV. Statistical analysis was performed with SPSS 15. Odds ratios (ORs) and 95% confidence intervals (CIs) were calculated.

Risk factors significantly associated with secondary cancers in univariate analyses were entered into a multivariate logistic regression model. Significance was set at p<0.05.

The vast majority of patients had early-stage disease: 64 (45.35%) stage IA, 30 (20.97%) stage IB, 24 (16.78%) stage IIA, 13 (9.09%) stage IIB, 4 (2.79%) stage IIIA, 3 (2.09%) stage IIIB, 4 (2.79%) stage IVA, and 1 (1.43%) stage IVB.

Stage IV disease, the presence of lymphomatoid papulosis, and duration of disease (more than 10 years) were shown to be the factors that increased the risk of developing secondary solid tumors (OR: 21.958, 95% CI: 2.039-839.657; OR: 19.926, 95% CI: 2.387-166.362; OR: 0.635, 95% CI: 0.420-0.959, respectively). In the vast majority of the patients, secondary malignancies occurred during the first year of diagnosis of MF (60%).

Our study supports previous findings about an increased risk of developing a second primary malignancy, especially Hodgkin lymphoma, chronic leukemia, and lung cancer, in patients with MF. In previous epidemiological studies, patients with MF had an elevated risk of secondary neoplasms (mean relative risk: 1.73, range: 1.32-2.4) [2 ,3]. Some authors have suggested that anti-lymphoma drugs [4] and particularly alkylating agents may lead to leukemia [4]. MF and hematological malignancies may have the same genetic origin, carcinogens, or viruses that affect lymphocyte precursors, and additionally the production of cytokines by the first neoplasm may induce the development of the secondary neoplasm [5]. It was shown that MF is a T helper cell 2 (Th2) mediated disease and is associated with human leukocyte antigen 2 alleles. The antigens causing inappropriate antigens presenting to T lymphocytes are still unknown. Viruses (Epstein-Barr virus, herpes simplex virus), deficiency of vitamin D, and medications are possible causative agents. In addition to these factors, increased levels of transforming growth factor-β, interleukin-10, and Th2 cytokines and the activation of STAT-3 oncogenes make the host immunosuppressed. We found that older age, stage of MF, and the presence of lymphomatoid papulosis increased the risk of coexistence of two other malignancies besides MF. Therefore, extensive evaluation for secondary malignancies in the adult population would be warranted, particularly if the patient has lymphomatoid papulosis.

## Figures and Tables

**Table 1:**
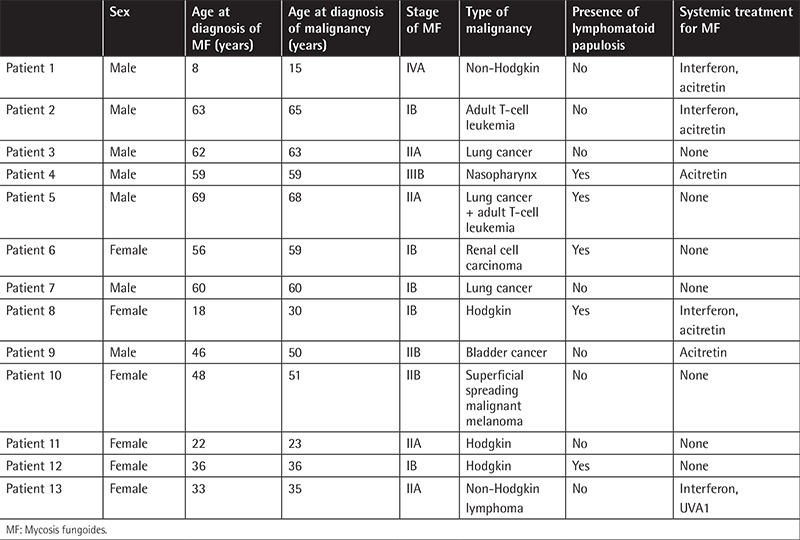
Clinical features of mycosis fungoides patients with secondary malignancies.
